# 3D bioprinting of tissue-specific osteoblasts and endothelial cells to model the human jawbone

**DOI:** 10.1038/s41598-021-84483-4

**Published:** 2021-03-01

**Authors:** Anna-Klara Amler, Alexander Thomas, Selin Tüzüner, Tobias Lam, Michel-Andreas Geiger, Anna-Elisabeth Kreuder, Chris Palmer, Susanne Nahles, Roland Lauster, Lutz Kloke

**Affiliations:** 1 Cellbricks GmbH, Gustav-Meyer-Allee 25, 13355 Berlin, Germany; 2 Department of Medical Biotechnology, Technische Universität Berlin, Gustav-Meyer-Allee 25, 13355 Berlin, Germany; 3 Department of Oral- and Maxillofacial Surgery, Charité Campus Virchow, Augustenburger Platz 1, 13353 Berlin, Germany

**Keywords:** Tissue engineering, Bone, Biomineralization, Tissues, Biomaterials - cells, Gels and hydrogels

## Abstract

Jawbone differs from other bones in many aspects, including its developmental origin and the occurrence of jawbone-specific diseases like MRONJ (medication-related osteonecrosis of the jaw). Although there is a strong need, adequate in vitro models of this unique environment are sparse to date. While previous approaches are reliant e.g. on scaffolds or spheroid culture, 3D bioprinting enables free-form fabrication of complex living tissue structures. In the present work, production of human jawbone models was realised via projection-based stereolithography. Constructs were bioprinted containing primary jawbone-derived osteoblasts and vasculature-like channel structures optionally harbouring primary endothelial cells. After 28 days of cultivation in growth medium or osteogenic medium, expression of cell type-specific markers was confirmed on both the RNA and protein level, while prints maintained their overall structure. Survival of endothelial cells in the printed channels, co-cultured with osteoblasts in medium without supplementation of endothelial growth factors, was demonstrated. Constructs showed not only mineralisation, being one of the characteristics of osteoblasts, but also hinted at differentiation to an osteocyte phenotype. These results indicate the successful biofabrication of an in vitro model of the human jawbone, which presents key features of this special bone entity and hence appears promising for application in jawbone-specific research.

## Introduction

Over the past decades, tissue engineering has made astounding progress in developing physiological models of human tissues with ever-growing sophistication and complexity. Particularly 3D bioprinting, the adaptation of 3D printing to the requirements for direct fabrication of living biological constructs, represents one of the latest achievements on the path to more physiological and therefore adequate models of the human body and manufacturing of customised tissue implants.

The emergence of this versatile technology has opened new possibilities for site-specific arrangement of multiple cell types combined with the production of delicate features embedded within structures of any possible geometry^1–5^. This allows for the implementation of vascularisation in fabricated tissue models, which is a mandatory element of tissue structure due to the biological requirement for nutrient and oxygen supply as well as waste product removal^6–8^.

The fabrication of bone-like tissue has been one of the most prominent research fields of tissue engineering in recent years; this is only partly accounted for by the striking clinical relevance^9–12^. In contrast to bone grafting, which aims at replacing or restoring functional bone in patients, bone models can be used to generate deeper insights into physiological and pathological processes. They are important for the development of novel therapeutic strategies and for reducing or replacing animal experiments. As an example, in vitro models for bone remodelling and associated disorders like osteoporosis have been previously established, as has been reviewed by Owen and Reilly^13^. These models naturally are simplified versions of the in vivo situation and therefore mimic only certain aspects.

While bone models are constantly being improved, it must be considered that craniofacial bones differ from other bones. Not only, jawbones show a higher turnover compared to other bone entities^14,15^, but they also arise from a distinct germ layer, originating in the neural crest in contrast to other bones emerging from the mesoderm^16^. In maxillofacial research, there is a strong need for jawbone models resembling key features of this unique bone entity to advance investigation of jawbone-specific diseases like MRONJ, a condition for which molecular details of pathogenesis as well as optimal treatment have yet to be unravelled^17–19^.

Models of the human jawbone in healthy and pathological condition are currently sparse, as most of the studies are performed using animal models, raising difficulties in transfer due to interspecies differences^20–22^. Penolazzi et al*.* established a three-dimensional co-culture system of osteoclasts and jawbone-derived osteoblasts based on aggregates, using cells from native and necrotic human bone tissue^23^. Similarly, a scaffold-based system combining alveolar bone and gingival tissue was developed by Almela et al*.* and employed as a model for oral cancer^24,25^. Other human jawbone models are also dependent on devices like scaffolds and microchip technologies^26–28^. To our knowledge, there have been no studies so far using 3D bioprinting for direct fabrication of human jawbone models.

In the present study, we demonstrate bioprinting of human jawbone models containing primary human jawbone-derived osteoblasts using projection-based stereolithography, which display key characteristics of jawbone. Optionally, fabricated channel structures within the constructs comprised primary endothelial cells to demonstrate the possibility to implement another cell type, potentially generating an even more sophisticated model. Bioprinted constructs were cultivated for 4 weeks and exhibited mineralisation of the matrix, while also showing expression of osteogenic marker genes and proteins, hinting at osteocyte differentiation.

## Results

### Bioprinting and cultivation of jawbone models

**Figure 1:**
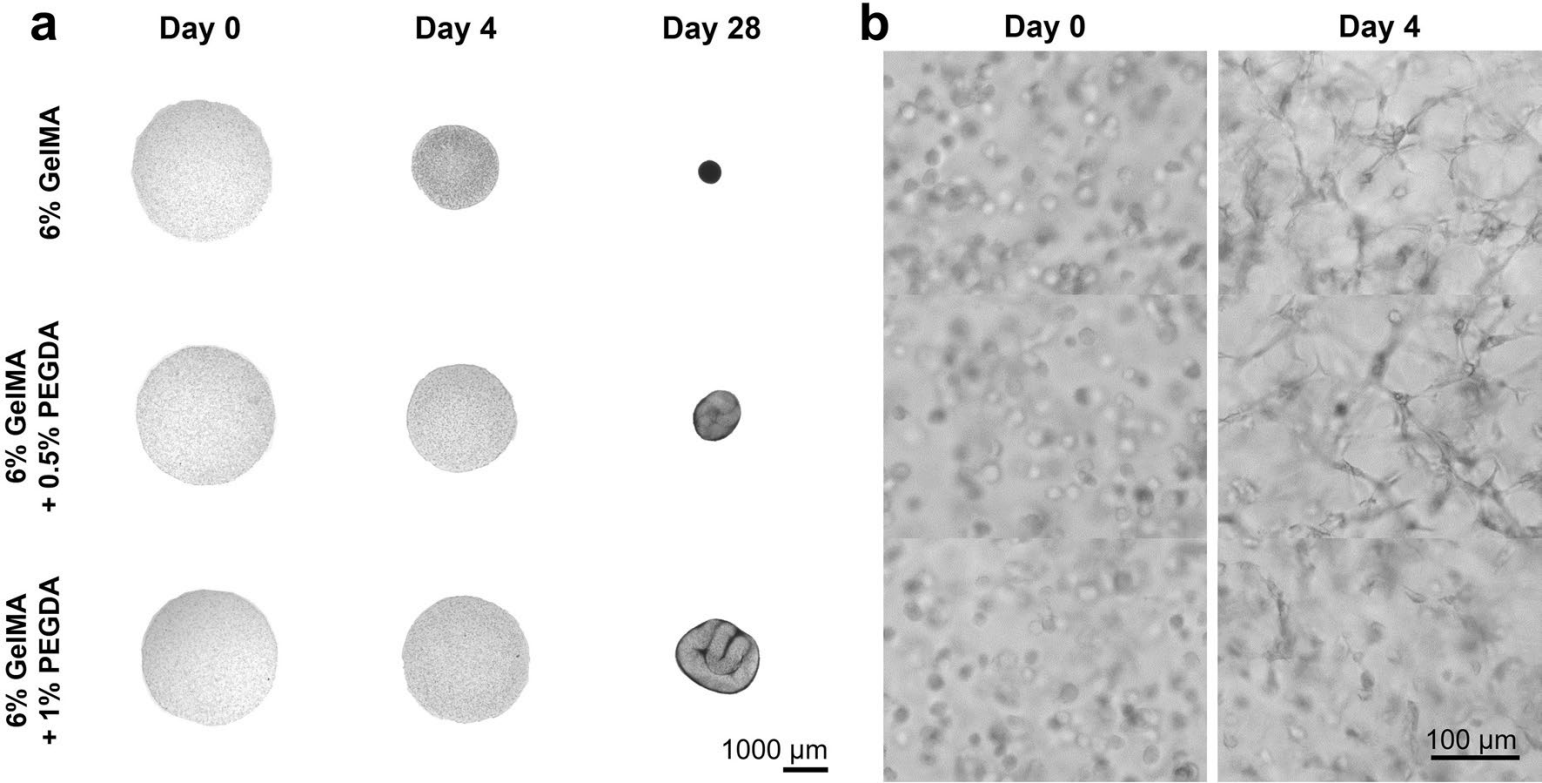
Material testing for fabrication of human jawbone models. (**a**) Dimensional stability of the printed constructs was dependent on the ink composition. (**b**) Cell spread of embedded osteoblasts was observed in all conditions after 4 days.

Human jawbone models were fabricated using the multi-material stereolithographic bioprinter developed by Cellbricks GmbH. First, to ensure dimensional stability of printed constructs, different ink compositions were tested for embedding primary jawbone-derived human osteoblasts (JHOBs) (Fig. 1). For stability tests, discs with 3 mm diameter and 0.5 mm height containing 20 × 10^6^ cells mL^−1^ were printed and cultivated in growth medium for 28 days. Utilisation of 6% GelMA resulted in quick and distinct contraction of the hydrogel, while supplementation with PEGDA3400 (0.5% and 1%) led to improved stability and therefore less contraction (Fig. 1a). As an indicator for material compatibility, cell spreading was observed to a similar degree for 6% GelMA and 6% GelMA + 0.5% PEGDA3400 after 4 days of cultivation, while it was less pronounced for 6% GelMA + 1% PEGDA3400 (Fig. 1b). 6% GelMA + 0.5% PEGDA3400 was identified as the favourable ink composition, as it combined support of cell function as well as hydrogel stability.

**Figure 2:**
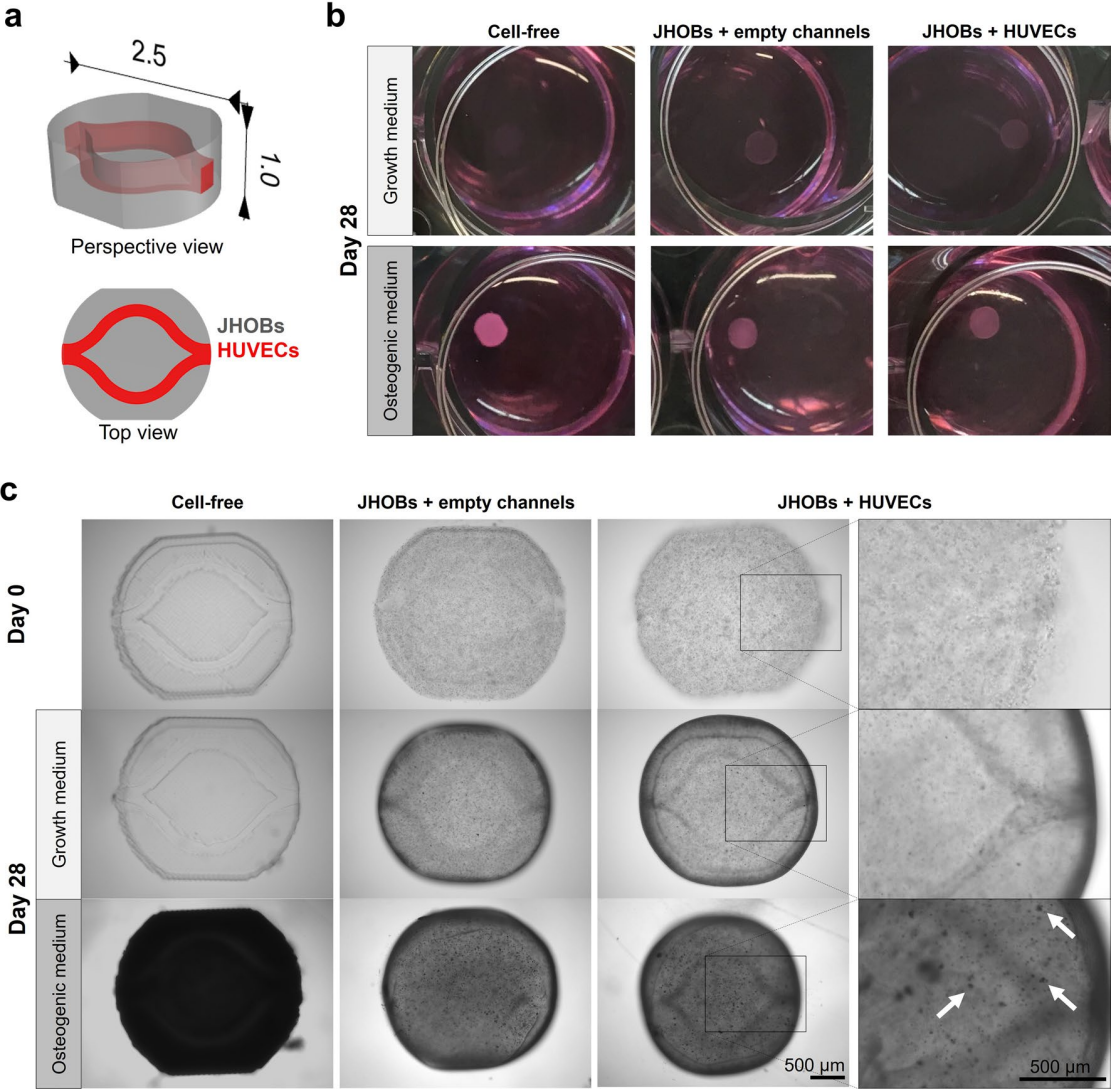
Fabrication and cultivation of the bioprinted human jawbone models. (**a**) CAD file of the jawbone model. Dimensions are given in mm. (**b**) Macroscopic images of the prints after 28 days of cultivation. (**c**) Microscopic images of the prints on day 0 and after 28 days of cultivation. White arrows indicate deposited nodules.

JHOBs embedded in 6% GelMA + 0.5% PEGDA3400 constitute the bulk part of the jawbone model (Fig. 2a, shown in grey), which is traversed by a channel, optionally containing human umbilical vein endothelial cells (HUVECs) (shown in red). Constructs without cells (“Cell-free”), with JHOBs (“JHOBs + empty channels”) as well as comprising both cells types in the respective structure (“JHOBs + HUVECs”) were successfully printed and cultivated for 28 days, both in growth and osteogenic medium. To fabricate constructs containing endothelial cells, HUVECs were bioprinted within a hyaluronic acid-based bioink to form cell-laden channels after treatment with hyaluronidase, as described by Thomas et al*.*^8^. To assure biocompatibility of this system, originally established with HUVECs only, with JHOBs used in the present study, monolayer cultures were incubated with hyaluronidase and viability was assessed after 20 h. Supplementation of medium with hyaluronidase did not affect cell viability of osteoblasts (P = 0.8723, Supplementary Fig. S1).

The size of cell-laden bioprints was found to diminish slightly over the 28 days of cultivation relative to cell-free constructs, which retained their shape. Constructs cultivated in osteogenic medium macroscopically appeared white after 28 days (Fig. 2b), which microscopically manifested as formation of small dark nodules (cell-laden, marked by white arrows) or uniform darkening (cell-free constructs) (Fig. 2c).

### Mineralisation

**Figure 3:**
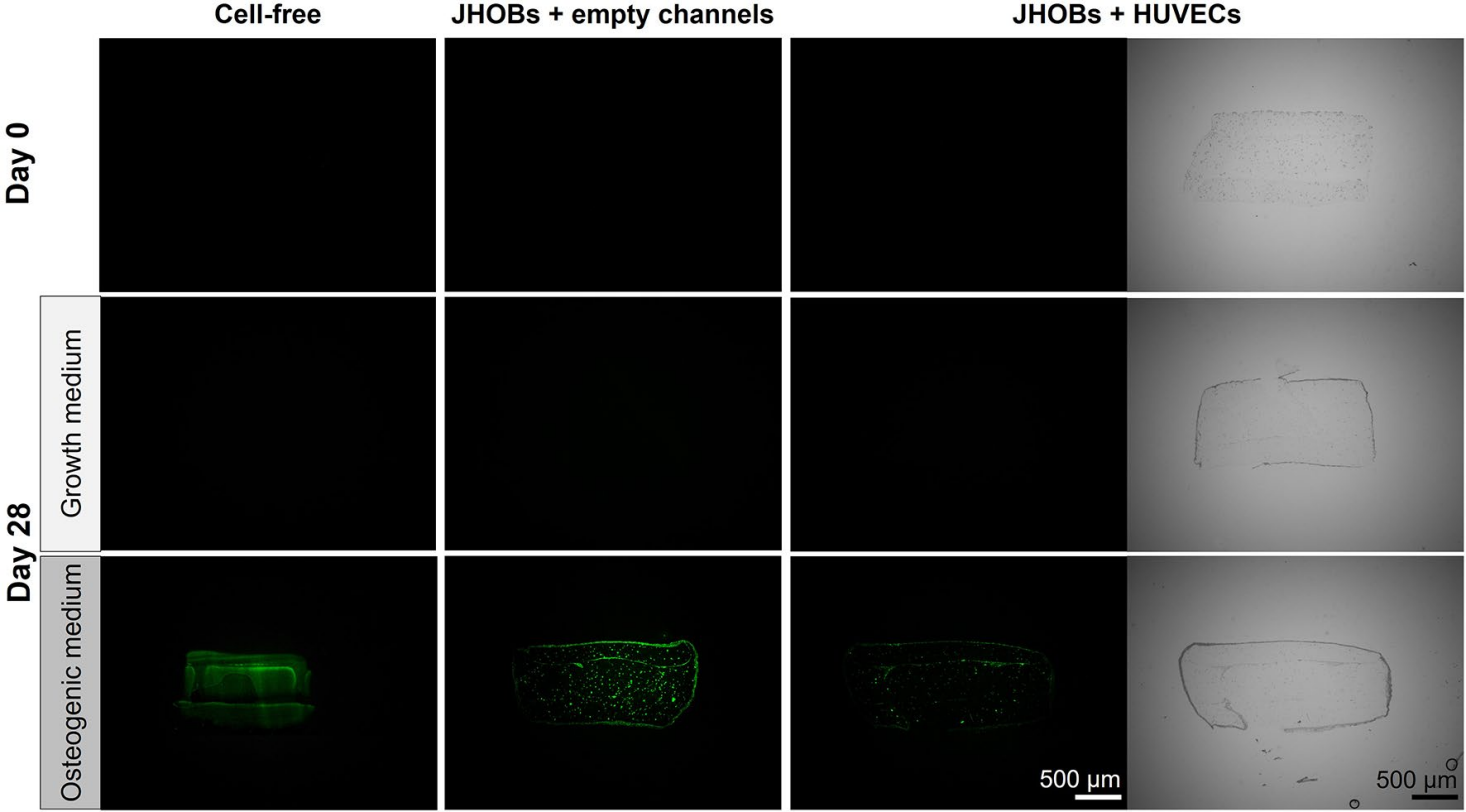
Mineralisation of the bioprinted human jawbone models. Histological staining of the prints on day 0 and day 28, using OsteoImage Mineralization Assay. For localisation of the sections, representative brightfield images are shown for the JHOBs + HUVECs condition.

Deposition of mineralised nodules is one of the key characteristics of osteoblasts, eventually leading to complete mineralisation of the bone matrix. In this process, nanoscale calcium phosphate crystals are incorporated into the previously secreted, soft matrix of the bone consisting mostly of collagen I fibrils, known as the osteoid^14,29,30^. To evaluate mineral formation within printed constructs, OsteoImage Mineralization Assay staining of cryosections was employed, which specifically stains the hydroxyapatite portion of deposited minerals. On day 28, nodule-like structures were detected throughout the prints in both JHOBs-containing conditions cultured in osteogenic medium, while cell-free constructs in osteogenic medium showed a distinct uniform staining pattern of the hydrogel (Fig. 3). Prints cultured in growth medium on day 28 showed no signal, and neither did all conditions on day 0.

### Gene expression

Gene expression of the bioprinted constructs was analysed using quantitative real-time PCR to assess osteogenic differentiation of the printed osteoblasts. Relative mRNA expression was normalised to the housekeeping gene ubiquitin-conjugating enzyme E2 D2 (*UBE2D2*). Runt-related transcription factor (*RUNX2*) and transcription factor Sp7 (*SP7*, also known as osterix) are two key regulators of osteogenic differentiation, while Sp7 is acting downstream of RUNX2, which is counted as one of the first osteoblast differentiation markers^13,16,31^. *ALPL* encodes for the membrane-bound enzyme alkaline phosphatase associated with bone mineralisation by hydrolysing pyrophosphate and providing inorganic phosphate^32^. Collagen I makes up the vast majority of organic compounds of the bone matrix and is, like ALPL, considered an early bone differentiation marker, with collagen type I alpha 1 chain (*COL1A1*) being the predominant collagen^33^. *SPARC* encodes for the ubiquitously expressed protein osteonectin involved in bone mineralisation, and is regarded as a late differentiation marker^26^. *DMP1* (dentin matrix acidic phosphoprotein 1), one of the SIBLING (small integrin-binding ligand, N-linked glycoprotein) proteins, is often employed as an early osteocyte marker^34,35^.

**Figure 4:**
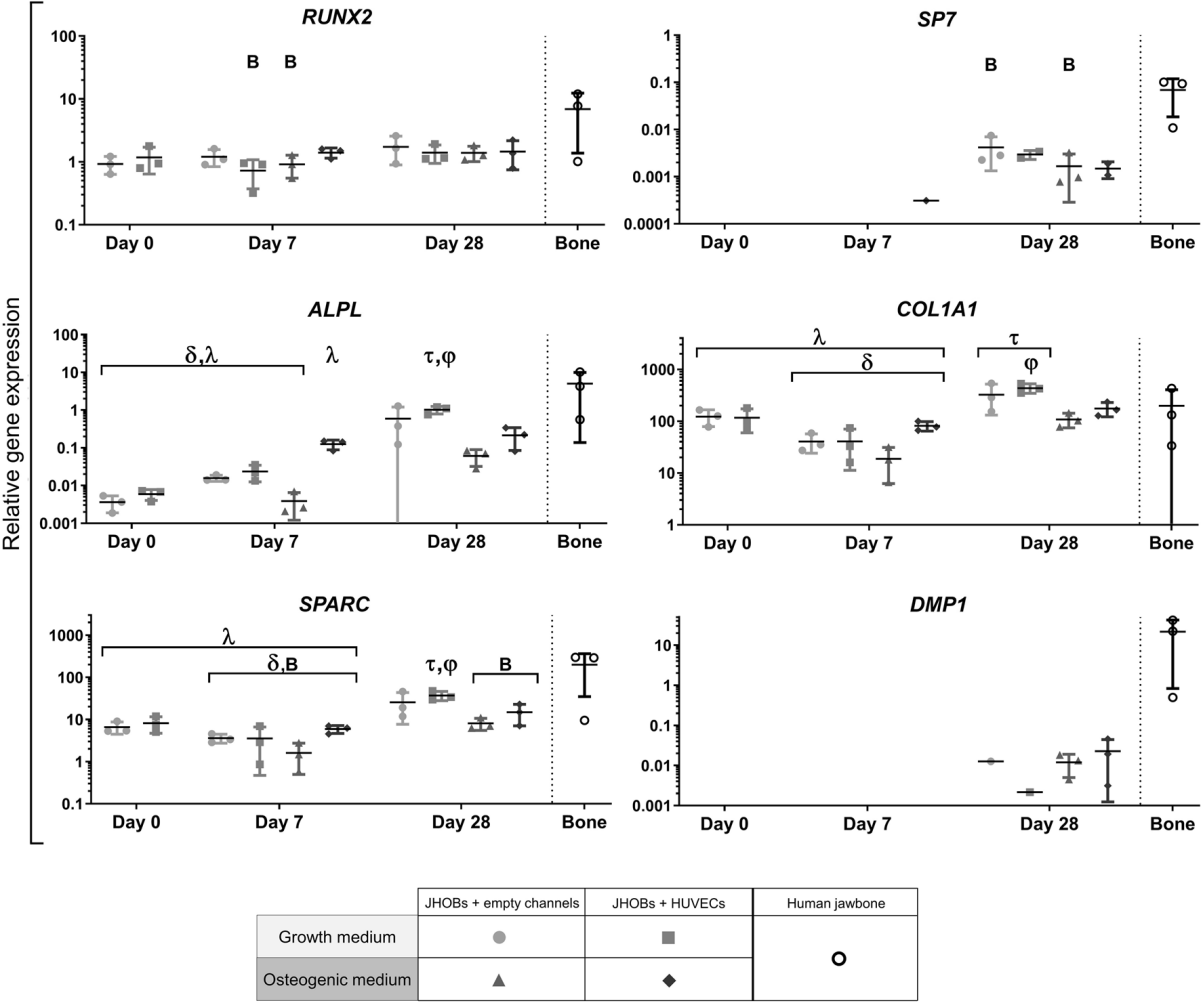
Marker gene expression of the bioprinted human jawbone models. Relative gene expression of the osteoblast differentiation markers *RUNX2* (Runt-related transcription factor 2), *SP7* (transcription factor Sp7/osterix), *ALPL* (alkaline phosphatase), *COL1A1* (collagen type I alpha 1 chain), *SPARC* (osteonectin) and *DMP1* (dentin matrix acidic phosphoprotein 1) in cell-laden constructs containing JHOBs and optionally also HUVECs after 0, 7 and 28 days of cultivation in growth and osteogenic medium. Native human jawbone was used as a control. Expression was normalized to *UBE2D2* expression. B = data significantly different to native human jawbone (P < 0.05). δ = data significantly different to JHOBs + empty channels in growth medium on day 28. λ = data significantly different to JHOBs + HUVECs in growth medium on day 28. τ = data significantly different to JHOBs + empty channels in osteogenic medium on day 28. φ = data significantly different to JHOBs + HUVECs in osteogenic medium on day 28. Data are presented as mean ± standard deviation. n = 3.

*RUNX2* expression remained stable at a level which did not deviate significantly from native jawbone except for JHOBs + HUVECs in growth medium and JHOBs in osteogenic medium on day 7 (Fig. 4). Differences between jawbone models with and without HUVECs were apparent for the expression of late osteogenic differentiation marker *SP7*. Here, “JHOBs + empty channels”-conditions showed significantly distinct expression levels to native bone control (P = 0.0434 for growth medium and P = 0.0363 for osteogenic medium), whereas prints additionally containing HUVECs did not. An upregulation of *ALPL* expression was observed for all conditions over the course of the experiment, where prints cultivated in osteogenic medium showed slightly lower expression levels compared to their counterparts in growth medium on day 28. *COL1A1* and *SPARC* exhibited similar expression patterns with a slight decrease on day 7 followed by an increase on day 28, again showing slightly reduced expression of osteogenic media samples compared to growth media conditions. *COL1A1* was expressed at a high level comparable to the native bone control for all samples, while *SPARC* mRNA levels differed significantly to the native jawbone control on day 7 for all conditions (P values: JHOBs in growth medium = 0.0273; JHOBs + HUVECs in growth medium = 0.0273; JHOBs in osteogenic medium = 0.0259; JHOBs + HUVECs in osteogenic medium = 0.0291) and on day 28 for osteogenic conditions only (P values: JHOBs = 0.0323; JHOBs + HUVECs = 0.0389). Similar to *SP7*, expression of *DMP1* was only detected after 28 days of cultivation, independent of the cell type composition and cultivation media, although a signal was detectable only for one of three replicates in each growth media condition.

### Histological analysis

**Figure 5:**
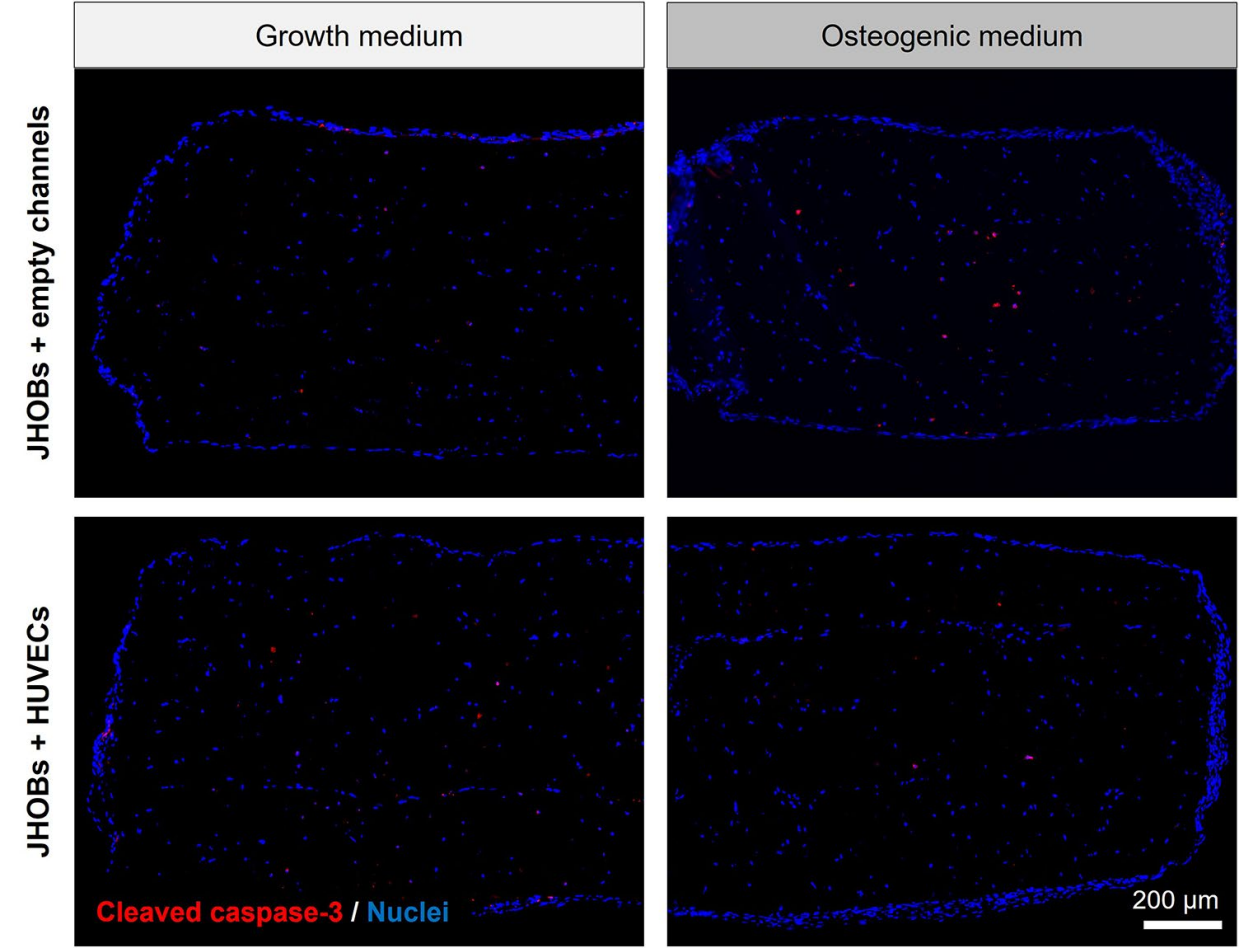
Immunohistological staining for apoptotic cells. Cell-laden constructs containing JHOBs and optionally HUVECs were stained for cleaved caspase-3 after 28 days of cultivation in growth and osteogenic medium.

To check for apoptotic cells, bioprinted jawbone models were cryosectioned and stained for cleaved caspase-3 after 28 days of cultivation (Fig. 5). A few cells positive for cleaved caspase-3 were detected for all conditions, being scattered throughout the constructs.

**Figure 6:**
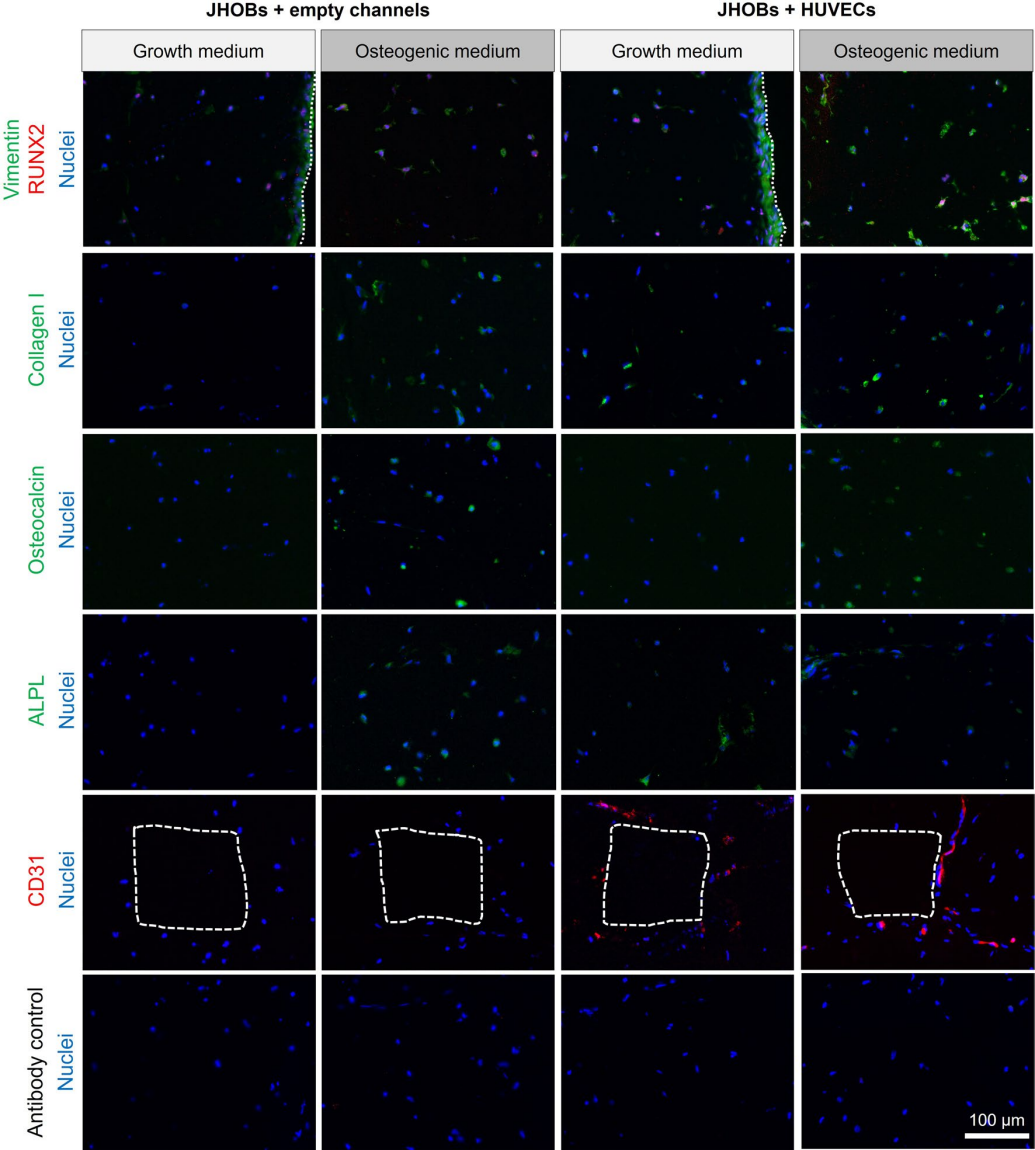
Immunohistological staining of the bioprinted human jawbone models. Cryosections were stained for osteoblast (RUNX2 (Runt-related transcription factor 2), collagen I, osteocalcin, ALPL (alkaline phosphatase)) and endothelial cell markers (CD31), in cell-laden constructs containing JHOBs and optionally also HUVECs after 28 days of cultivation in growth and osteogenic medium. Channels are marked with white dashed lines (CD31 staining), and the surface of the constructs is marked with white dotted lines.

Immunohistological staining was also performed to confirm osteoblast and endothelial cell marker expression on the protein level and to visualise their localisation (Fig. 6). All cell-laden conditions exhibited staining for osteoblast marker protein RUNX2 on day 28, which colocalised with DAPI-stained nuclei as expected, since it acts as a transcription factor during osteoblast differentiation. Collagen I and ALPL were detected in all conditions except in constructs containing only JHOBs and cultured in growth medium. Staining of vimentin revealed a monolayer covering the surface of the printed models, resulting from proliferation of cells not fully incorporated into the print matrix.

Osteocalcin is solely expressed by osteoblasts and is one of the most abundant proteins in bone beside collagen I^36,37^. In this study, osteocalcin was detected after 28 days throughout constructs cultivated in osteogenic medium, while no expression was observed in the growth medium conditions. Presence of CD31-positive cells after cultivation for 28 days was shown in both JHOBs + HUVECs conditions, indicating endothelial cell phenotype being supported by osteoblasts. CD31 staining was not exhibited by constructs containing only JHOBs, as anticipated (Fig. 6). Cell-free constructs did not stain for any of the marker proteins (Supplementary Fig. S2).

## Discussion

In the present study, we successfully fabricated physiological jawbone models featuring a vasculature-like structure via stereolithographic bioprinting. GelMA, a hydrogel based on natural extracellular matrix, was chosen as the main component of the bioink as it represents a natural source of RGD sequences and can be modified by cells^38–40^. Printing of discs composed of 6% GelMA containing jawbone-derived osteoblasts resulted in strong shrinkage of the hydrogel, already observable after four days, where cells displayed an elongated morphology (Fig. 1). Spreading of cells is generally valued as favourable and usually occurs in less stiff matrices^41–43^, while Duarte Campos et al*.* even demonstrated that enhanced cell spreading within hydrogels coincided with enhanced osteogenic differentiation of mesenchymal stromal cells^41^. To improve hydrogel stability while simultaneously maintaining cell-friendly properties, two combinations of GelMA with PEGDA3400 (0.5% and 1%) were tested. Addition of PEGDA resulted in enhanced resistance to shrinkage, but seemingly compromised cell spreading at higher concentrations as it was less pronounced in hydrogels consisting of 6% GelMA + 1% PEGDA3400. These findings both point towards increased stiffness of mixed hydrogels^42,44^. Da Branco et al*.* proposed the existence of a certain threshold of matrix stiffness for fibroblasts embedded in alginate/collagen mixtures to be able to spread within and contract the hydrogel^42^. If such a threshold exists for our system, stiffness of the tested conditions was below this as contraction still occurred to some degree. 6% GelMA + 0.5% PEGDA combined both improved hydrogel stability and support of cell spreading, and was therefore chosen as the bioink for printing jawbone models. Contraction of these models was hardly observable, possibly due to their shape, since simple discs were used for material testing, whereas jawbone models possessed a more complex architecture and a higher aspect ratio (Fig. 2).

One of the current key challenges in tissue engineering is the construction of vasculature-like structures to overcome diffusion limits of nutrients and oxygen, allowing for removal of waste products and therefore enabling creation of tissue structures potentially serving as transplant material. Thomas et al*.* established a method to fabricate delicate channel structures lined with HUVECs for use with stereolithographic bioprinters. Briefly, channels were printed by light-induced solidification of 1.5% HAMA + 0.05% LAP, simultaneously embedding endothelial cells suspended in the ink. Subsequent digestion of the hyaluronic acid-based hydrogel using hyaluronidase resulted in release and attachment of the cells to the channel walls. This procedure did not affect viability of HUVECs^8^. Similarly, no adverse effect of the hyaluronidase treatment on JHOBs was found in our study (Supplementary Fig. S1), paving the way for 3D bioprinting of vascularised jawbone models. Printed channel structures were well visible (Fig. 2c). After 28 days of cultivation, cells growing in the channels still stained positive for CD31 in both growth and osteogenic media, demonstrating survival of printed HUVECs in co-culture with embedded osteoblasts without further supplementation of the medium with growth factors normally used for endothelial cell culture (Fig. 6). Jawbone models cultivated in growth medium and containing HUVECs showed expression of collagen I and ALPL, while this was not detected for constructs in the same medium without HUVECs, indicating that the co-culture promotes osteoblast differentiation as has been described before^45,46^. Pre-differentiation of osteoblasts prior to fabrication of jawbone prints might improve the results seen here even further by promoting network formation of endothelial cells^45,47^. These studies utilised osteogenic medium mixed with endothelial growth medium, which led to better survival of HUVECs^45^. Therefore, optimisation of medium composition could enhance network formation. However, we already observed HUVEC survival in media only based on DMEM, without requiring endothelial growth medium supplemented with growth factors.

Calcification of the surrounding matrix by osteoblasts is crucial for the generation of hard bone tissue. Here, mineralisation of printed jawbone models cultivated in osteogenic medium was demonstrated, whereas prints cultivated in growth medium did not display any mineralisation. This was already macroscopically observable as a white staining of the prints in osteogenic medium, while the constructs in growth medium remained transparent after 28 days in culture (Fig. 2b). Cell-laden constructs in growth medium usually appear slightly opaque due to the embedded cells. Furthermore, the structure of the deposited mineralisation was visualised on cryosections using the OsteoImage Mineralization Assay specifically staining the hydroxyapatite portion of minerals (Fig. 3). Both osteoblast-featuring conditions displayed formation of nodule-like structures distributed through the construct, presumably nucleation sites needed for propagation of the mineralisation, which are induced by matrix vesicles containing hydroxyapatite crystals and budding from the surface of the cells^32^. Park et al*.* reported mineralisation of osteogenically differentiated periosteal cells as early as day 14, being in accordance with our observations^48^. In contrast, cell-free constructs exhibited a uniform staining of the print matrix. Mineralisation of cell-free hydrogels was previously described by Klouda et al*.*, who stated that medium supplemented with fetal calf serum (FCS), as used in this study, facilitated occurrence of this process. This might be due to the composition of FCS including, amongst other components, proteins acting as calcification factors or featuring an endogenous ALP activity. They also reported earlier detection of mineralisation in cell-laden constructs compared to cell-free hydrogels, coinciding with our observations^49^.

Osteocalcin is upregulated in matrix synthesis and mineralisation as it acts as a regulator via binding of calcium apatite, and is considered a late osteoblast differentiation marker^25,37,50–52^. Its expression in prints cultivated in osteogenic medium was demonstrated by immunohistological staining (Fig. 6), starting as early as day 14. This is in accordance with Park et al*.*, who reported osteocalcin secretion by periosteal-derived cells after 14 days of osteogenic induction^48^. Due to the osteocalcin expression, mineralisation of cell-laden constructs might take place in a more directed manner than in the cell-free conditions, where osteocalcin was not detected (Supplementary Fig. S2). While mineralisation of hydrogels containing primary osteoblasts as well as osteoblast-like cell lines has been reported as early as after 7 days^53,54^, Wein et al*.* demonstrated a lower mineralisation capacity of osteoblasts originating from alveolar bone compared to iliac crest-derived bone from the same donor^55^. Hence, prolonged incubation times could enable advanced calcification of the printed jawbone models presented here^28^.

RNA samples were taken from the whole construct, resulting in measurement of the expression levels of both cell types in conditions containing both JHOBs and HUVECs. Gene expression was normalised to *UBE2D2* expression, which is also found in HUVECs. This might result in lower relative expression of osteogenic marker genes in comparison to constructs containing no HUVECs, which should be kept in mind when directly comparing “JHOBs + empty channels” to “JHOBs + HUVECs”. However, cells were introduced at a ratio of 9:1 (JHOBs:HUVECs), thus putatively having only a small impact on overall expression levels. Furthermore, osteoblasts have been shown to present a heterogenous phenotype^56^. Due to the small sample size, the native bone samples used for quantitative gene expression analysis were not taken from the same donor used for the bioprinting experiments. The three tested donors showed high variability in all osteogenic markers, resembling the natural interindividual differences. As a result, statistically significant differences were only determined for some samples.

Osteocytes share similar markers with osteoblasts, except that they display no expression of ALPL^35^. In contrast, they express DMP1, which is considered one of the early osteocyte markers^35,57^. Expression of *DMP1* was detected only after 28 days of cultivation in all conditions. In osteogenic medium, all replicates were positive for *DMP1,* while in growth medium only one replicate in each condition was positive. This points towards differentiation of printed osteoblasts to osteocytes, although expression levels are still low compared to native jawbone. Osteogenic medium promoted this tendency. In addition, the 3D environment itself already seemed to be osteoinductive since *DMP1* was also detected for growth medium conditions without osteogenic supplements (Fig. 4)^58^. Similar observations were made for *SP7*, a transcription factor necessary for osteogenic differentiation and being expressed later than RUNX2^16^. The progress of osteogenic differentiation might also explain the lowered expression levels of *COL1, ALPL* and *SPARC* on day 28 in models in osteogenic medium compared to growth medium. Expression of these genes is normally increased during differentiation of MSCs to osteoblasts and is decreased again as cells move towards an osteocyte phenotype^11,25,27,58,59^. Longer cultivation of the printed jawbone models could enhance these observations. In contrast, expression levels of the transcription factor *RUNX2* remained stable in all conditions over the course of the experiment, also observed in the immunostaining (Fig. 6). This is explained by its function which is not only crucial for differentiation of osteoblasts, but also for maintaining their function, e.g. the synthesis of bone matrix^16,58,60^.

The model presented here resembles only certain characteristics of the human jawbone. Key features like mineralisation and gene expression of osteogenic markers are not distinguishable from long bones as such, since there are no jawbone-specific markers known up to now. Nevertheless, we are convinced that our model represents the human jawbone and not just any other bone entity. The specificity of the model lies not in a certain sophisticated geometry but is rather intrinsic to the implemented cells themselves. Marolt et al*.* summarised several studies concluding that there is indeed a physiological difference between osteogenic cells from jawbone versus long bones, making them mandatory for generation of a jawbone model^61^. In line with this, several jawbone models have been proposed based on the origin of the isolated osteoblasts^23,25,27,55,62^.

## Conclusion

For the first time, human jawbone models were fabricated via stereolithographic bioprinting. These models were comprised of primary human jawbone-derived osteoblasts embedded in the print matrix. Optionally, endothelial cells were included in vasculature-like 3D printed channel structures, showing the possibility to implement another cell type for increasing complexity of the model. Over the 28 days of cultivation, both cell types did not only survive and maintain their marker gene expression, but also indicated differentiation of JHOBs to osteocytes. As a next step, the applicability of our models for in vitro investigation of diseases like MRONJ should be tested. Longer cultivation of the printed constructs could enable further differentiation and lead to an even more physiological model of the human jawbone, since in vivo, mineralisation of bone takes a few months in the first phase, followed by long-term secondary mineralisation which includes maturation of the tissue^63^. Likewise, other cell types like osteoclasts could be implemented to improve the model further by increasing complexity^23,64^, as could dynamic cultivation by providing mechanical cues to the cells^26^. Of special interest is the establishment of a functional endothelium, opening the possibility to investigate angiogenesis-related aspects of MRONJ.

## Methods

### Photoink synthesis

Methacrylated gelatine (GelMA) was synthesised as described previously^65,66^. Briefly, Type A gelatine (300 bloom; Sigma-Aldrich, Saint Louis, USA) from porcine skin was dissolved at 10% w/v in phosphate-buffered saline (PBS) and heated to 50 °C. Methacrylic anhydride was added dropwise at 0.1 mL g^−1^ gelatine used, reaction was allowed for three hours under stirring and pH was adjusted to 7.4. GelMA was dialysed against distilled water through a 12–14 kDa cut-off membrane for four days to remove remaining salts and methacrylate. Afterwards, GelMA was lyophilised at − 60 °C and 1 mbar before being stored at − 20 °C until further usage.

Methacrylated hyaluronic acid (HAMA) was synthesised according to a modified protocol by Poldervaart et al*.*^67^ To reduce chain length, hyaluronic acid from *Streptococcus equi* (molecular weight > 1 MDa, Alfa Aesar, Ward Hill, USA) was autoclaved. Following this, hyaluronic acid (2.5 g) was dissolved in Milli-Q water (250 mL) and the solution was adjusted to pH 9.0 with NaOH (1 N). Methacrylic anhydride (5 mL) dissolved in dimethyl sulphoxide (5 mL) was added at 2 mL g^−1^ hyaluronic acid used and reaction was allowed for 24 h at room temperature under stirring. Following dialysis against Milli-Q water, HAMA was lyophilised and stored at − 20 °C until further usage. Methacrylation of synthesised GelMA and HAMA was verified by 1H-NMR using a Bruker Avance III at 500 MHz (Bruker Corporation, Billerica, USA).

Lithium phenyl-2,4,6-trimethylbenzoyl phosphinate (LAP) was synthesised as described previously^68,69^ and used as photoinitiator for polymerisation. Poly(ethylene glycol) diacrylate (MW 3400 Da, PEGDA3400) was purchased from Alfa Aesar (Ward Hill, USA). All synthesis reagents were purchased from Sigma-Aldrich (Saint Louis, USA) unless stated otherwise.

### Cell isolation and culture

Primary jawbone-derived human osteoblasts (JHOBs) were isolated following slightly modified protocols^36,55^. Briefly, small bone pieces (approximately 8 mm^3^) of human mandibular jawbone were obtained from healthy donors during surgery (as approved by Charité’s Ethics Committee, EA1/038/19; informed consent of all participating subjects was obtained and all methods were performed in accordance with the relevant guidelines and regulations) and stored in growth medium [high-glucose DMEM supplemented with 10% fetal calf serum, 100 IE mL^−1^ penicillin, 100 µg mL^−1^ streptomycin and 2 mM GlutaMAX (Gibco, Thermo Fisher Scientific, Waltham, USA)] at 4 °C until isolation. Jawbone pieces were rinsed thoroughly with PBS and soft tissue was removed using a scalpel. To reduce the germ load, samples were incubated in Betaisodona iodide solution (Mundipharma, Frankfurt am Main, Germany) for 60 s followed by repeated rinsing with PBS. Bone pieces were put in a 60 mm tissue culture-treated petri dish covered with growth medium. Cells were expanded upon confluency around the bone pieces and used at passage 5. Human umbilical vein endothelial cells (HUVECs) were cultured in Endothelial Cell Growth Medium 2 (PromoCell, Heidelberg, Germany) and used at passage 3. All consumables were obtained from Corning Inc. (Corning, USA) unless stated otherwise.

### Bioprinting and post-treatment

Three-dimensional models were designed using Rhinoceros 6 (Robert McNeel & Associates, Seattle, USA) and photomasks were generated using the bioprinter’s software. Photoinks were prepared by dissolution and subsequent dilution of the lyophilised material in PBS. To fabricate cell-laden constructs, JHOBs and HUVECs were mixed at 20 × 10^6^ cells mL^−1^ into the respective ink directly before printing. Bioprinting was performed using our proprietary stereolithographic printing platform as described previously^1,8^. Briefly, hydrogels were precisely solidified by photopolymerisation as photomasks were projected onto the printing dishes, and three-dimensional constructs were built by subsequent illumination of consecutive layers.

For material testing, inks were prepared according to Table 1 and discs with 0.5 mm height and 3 mm diameter were printed.

**Table 1:** Ink compositions for material testing.

Ink	GelMA (%)	LAP (%)	PEGDA3400 (%)
1	6	0.1	–
2	6	0.1	0.5
3	6	0.1	1

For jawbone models, 1.5% HAMA and 0.05% LAP were used as the channel ink, while the bulk material was printed using 6% GelMA, 0.5% PEGDA3400 and 0.1% LAP. Dimensions of the constructs were 2.6 mm × 2.4 mm × 1 mm. After completion of the printing process, constructs were incubated in 150 U mL^−1^ hyaluronidase (STEMCELL Technologies, Vancouver, Canada) in growth medium for 260 min to allow for enzymatical digestion of the printed channel structure and subsequent attachment of released HUVECs to the channel walls, adapted from Thomas et al*.*^8^

### Bioprint cultivation

For material testing, bioprints were cultivated in growth medium for 28 days in 24-well ultra-low attachment multiple well plates, while medium was exchanged three times a week.

For jawbone models, constructs were cultivated in growth medium or mineralisation medium [growth medium supplemented with 10 mM β-glycerophosphate (Sigma-Aldrich, Saint Louis, USA), 10 nM dexamethasone (AppliChem, Darmstadt, Germany) and 284 µM ascorbic acid phosphate (Sigma-Aldrich, Saint Louis, USA)] for up to 28 days in 24-well ultra-low attachment multiple well plates. Medium was exchanged three times a week.

Live imaging was performed throughout the cultivation periods using the BIOREVO BZ-9000 microscope (Keyence, Osaka, Japan).

### Biocompatibility assay for channel fabrication

To assess the biocompatibility of the bioprinting system regarding the effect of hyaluronidase on osteoblast cell viability, JHOBs were seeded into 96-well multiple well plates at a cell density of 1.25 × 10^4^ cells cm^−2^ and cultured in growth medium. The next day, medium was switched to growth medium supplemented with 150 U mL^−1^ hyaluronidase, or without as a control (n = 6, biological replicates from one donor). After further 20 h of cultivation, samples were stained with 5 µg mL^−1^ Hoechst33342 and 5 µg mL^−1^ propidium iodide (Sigma-Aldrich, Saint Louis, USA) in growth medium for 10 min at 37 °C and 5% CO2. Scans of each well were recorded using the Keyence BZ-9000 microscope, followed by digital stitching and analysis for numbers of Hoechst33342 and propidium iodide positive cells using the BZ-Analyzer II image analysis software (Keyence, Osaka, Japan).

### Histology

Printed constructs were washed in PBS, fixated with 4% paraformaldehyde (Electron Microscopy Sciences, Hatfield, USA) for 15 min at room temperature, washed in PBS, embedded in Tissue-Tek O.C.T. Compound (Sakura, Alphen aan den Rijn, The Netherlands), and incubated for 35 min at 37 °C. Following shock-freezing in liquid nitrogen, samples were stored at − 80 °C until further usage. 10 µm sections were produced using the CM1950 cryostat (Leica Microsystems, Wetzlar, Germany). Immunofluorescence staining was performed to evaluate expression of marker proteins. Following permeabilisation with acetone at − 20 °C for 10 min, samples were washed with PBS three times and blocked with 10% goat serum (Sigma-Aldrich, Saint Louis, USA) in PBS for 30 min at room temperature. Primary antibodies were diluted 1:100 in blocking buffer and incubated over night at 4 °C (rabbit anti-RUNX2, mouse anti-osteocalcin, mouse anti-alkaline phosphatase, rabbit anti-cleaved caspase-3 (Abcam, Cambridge, United Kingdom); mouse anti-collagen I (Sigma-Aldrich, Saint Louis, USA); mouse anti-vimentin (Santa Cruz Biotechnology, Dallas, USA); and mouse anti-CD31 (Invitrogen, Carlsbad, USA)). For antibody controls, samples were incubated with blocking buffer only. After washing three times with PBS, samples were incubated with secondary antibodies (goat anti-mouse-CF488 and goat anti-rabbit-CF594 (Biotium, Fremont, USA)) at a dilution of 1:200 in blocking buffer for 45 min at room temperature, and simultaneously counterstaining with 4′,6-diamidin-2-phenylindol (DAPI; Roche, Basel, Switzerland). Slides were washed three times with PBS and mounted using Imsol Mount (ImmunoLogic, Duiven, Netherlands). Images were taken using the BIOREVO BZ-9000 microscope (Keyence, Osaka, Japan).

### Mineralisation

To analyse mineralisation of printed constructs, OsteoImage Mineralization Assay (Lonza, Basel, Switzerland) was performed according to the manufacturer’s protocol. Samples were sectioned and permeabilised as described above. After 2 × washing with PBS and 1 × with wash buffer, sections were incubated with staining reagent for 30 min at room temperature. Slides were washed three times with wash buffer and mounted using Imsol Mount (ImmunoLogic, Duiven, Netherlands). Images were taken using the BIOREVO BZ-9000 microscope (Keyence, Osaka, Japan).

### Real-time PCR

Relative expression of bone-specific marker genes was assessed by semi-quantitative real-time PCR (see Table 2).

**Table 2:** Sequences of primers used for real-time PCR.

Gene	Description	For	Rev
*UBE2D2*	Ubiquitin-conjugating enzyme E2 D2	tcttgacaattcatttcccaacag	tcaggcactaaaggatcatctgg
*ALPL*	Alkaline phosphatase	cccacttcatctggaaccgc	ccgtggtcaattctgcctcc
*RUNX2*	RUNX family transcription factor 2	tcacaaatcctccccaagtagc	ggcgggacacctactctcatac
*COL1A1*	Collagen type I alpha 1 chain	gccgtgacctcaagatgtg	gccgaaccagacatgcctc
*SP7*	Sp7 transcription factor	tccatctgcctggctcctt	gttgttgagtcccgcagagg
*DMP1*	Dentin matrix acidic phosphoprotein 1	ttgtgaactacggagggtagagg	cctgagccaaatgacccttc
*SPARC*	Secreted protein acidic and rich in cysteine	gcagaagctgcgggtgaagaa	ctcgaaaaagcgggtggtgc

Total mRNA of printed jawbone models was isolated after 0, 7 and 28 days using the NucleoSpin RNA XS kit (MACHEREY–NAGEL, Düren, Germany) following the manufacturer’s protocol. For every condition and sampling time, three bioprinted constructs were analysed (n = 3, from one donor). For isolation of mRNA from native jawbone tissue, small bone pieces (approximately 8 mm^3^) were shortly rinsed with PBS and incubated with lysis buffer for 10 min followed by the usual isolation protocol of the same kit (n = 3, donors). mRNA was quantified using the NanoDrop 2000c spectrophotometer (Thermo Fisher Scientific, Waltham, USA) and cDNA was synthesised using the TaqMan Reverse Transcription kit (Applied Biosystems, Foster City, USA) according to the manufacturer’s protocol. Real-time PCR was performed using the Mx3005P Real-Time PCR system (Agilent Technologies, Santa Clara, USA). For this, 10 µM of the respective primers, cDNA equivalent to 10 ng total mRNA and SensiFAST SYBR No-ROX qPCR master mix (Bioline, Luckenwalde, Germany) were mixed in a total volume of 20 µL. Melting curve analysis was performed after each PCR run to exclude non-specific amplification. Relative mRNA expression of marker genes was normalised to the house-keeping gene ubiquitin-conjugating enzyme E2 D2 (*UBE2D2*).

### Statistical analysis

Statistical analyses were performed using GraphPad Prism 8 (San Diego, USA). All values are given as mean ± standard deviation. Two-way ANOVA with Tukey’s multiple comparison test was applied to real-time PCR data *for ALPL, COL1A1, RUNX2* and *SPARC* to check for significant changes between all print conditions. *DMP1* and *SP7* data were analysed using one-way ANOVA with Tukey’s multiple comparison test. One-way ANOVA with Dunnett’s multiple comparison test of all print conditions and native jawbone control was performed separately for each time point. Mann–Whitney test was used to analyse data of the biocompatibility test. P values smaller than or equal to 0.05 were considered significant.

## Supplementary Information

Supplementary Information
