# Supplementary material for: 3D bioprinting of tissue-specific osteoblasts and endothelial cells to model the human jawbone
Source: Sci Rep. 2021 Mar 1;11:4876. doi: 10.1038/s41598-021-84483-4 (PMC7921109; doi:10.1038/s41598-021-84483-4)
Supplement: Supplementary file 1 — Supplementary Information [file 41598_2021_84483_MOESM1_ESM.pdf]

3D bioprinting of tissue-specific osteoblasts and endothelial cells to model the human jawbone

Anna-Klara Amler[1,2], Alexander Thomas[1,2], Selin Tüzüner[1,2], Tobias Lam[1], Michel-Andreas Geiger[1], Anna-Elisabeth Kreuder[1,2], Chris Palmer[1], Susanne Nahles[3], Roland Lauster[2], Lutz Kloke[1]*

[1] Cellbricks GmbH, Gustav-Meyer-Allee 25, 13355 Berlin, Germany

[2] Technische Universität Berlin, Department of Medical Biotechnology, Gustav-Meyer-Allee 25, 13355 Berlin, Germany

[3] Department of Oral- and Maxillofacial Surgery, Charité Campus Virchow, Augustenburger Platz 1, 13353 Berlin, Germany

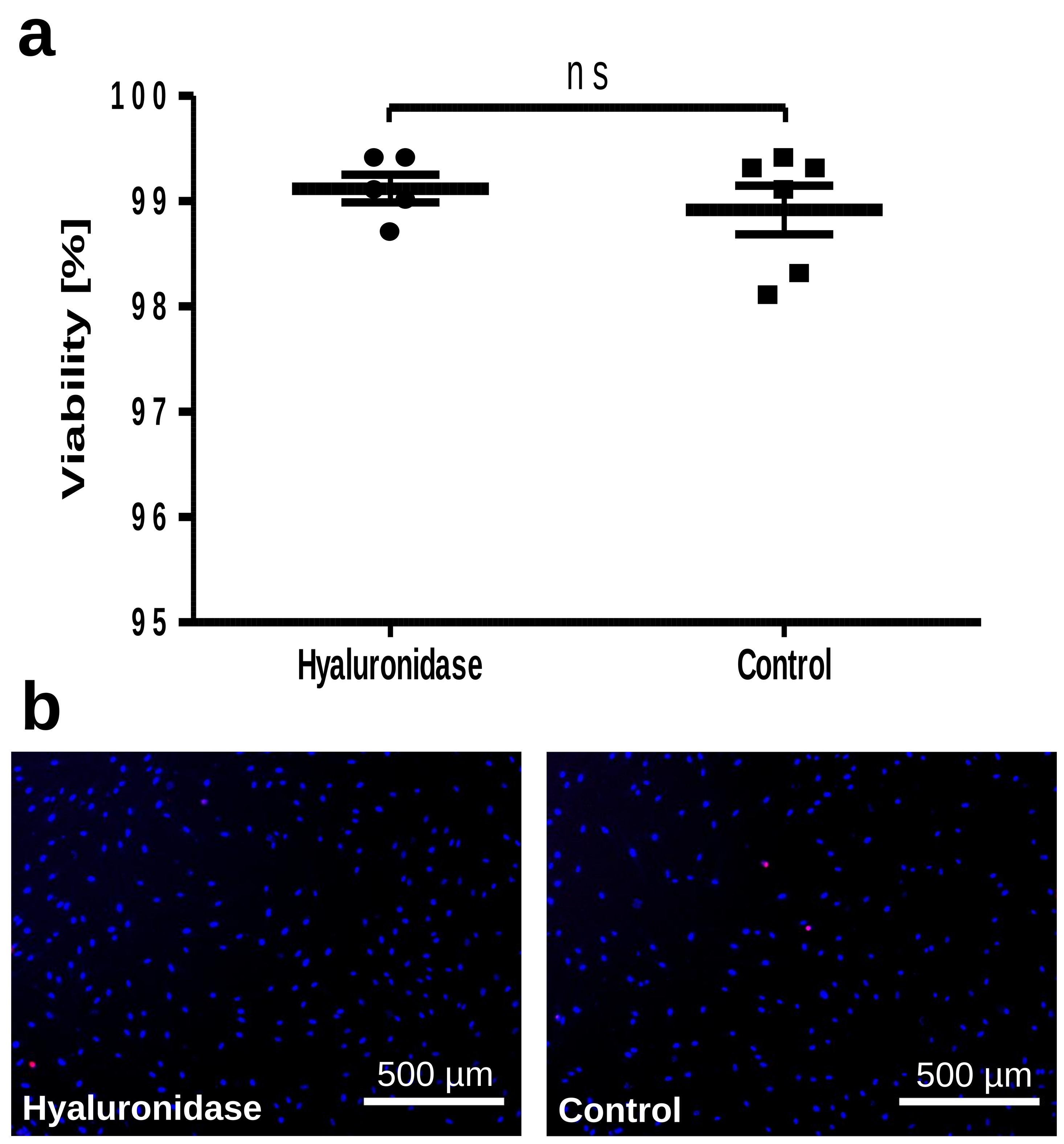

Supplementary Figure S1. Biocompatibility test. (a) Viability of osteoblasts after 20 hours cultured with and without hyaluronidase. Data are presented as mean ± standard deviation. (b) Representative images of stained osteoblasts cultured with (left) and without hyaluronidase (right). n = 6.

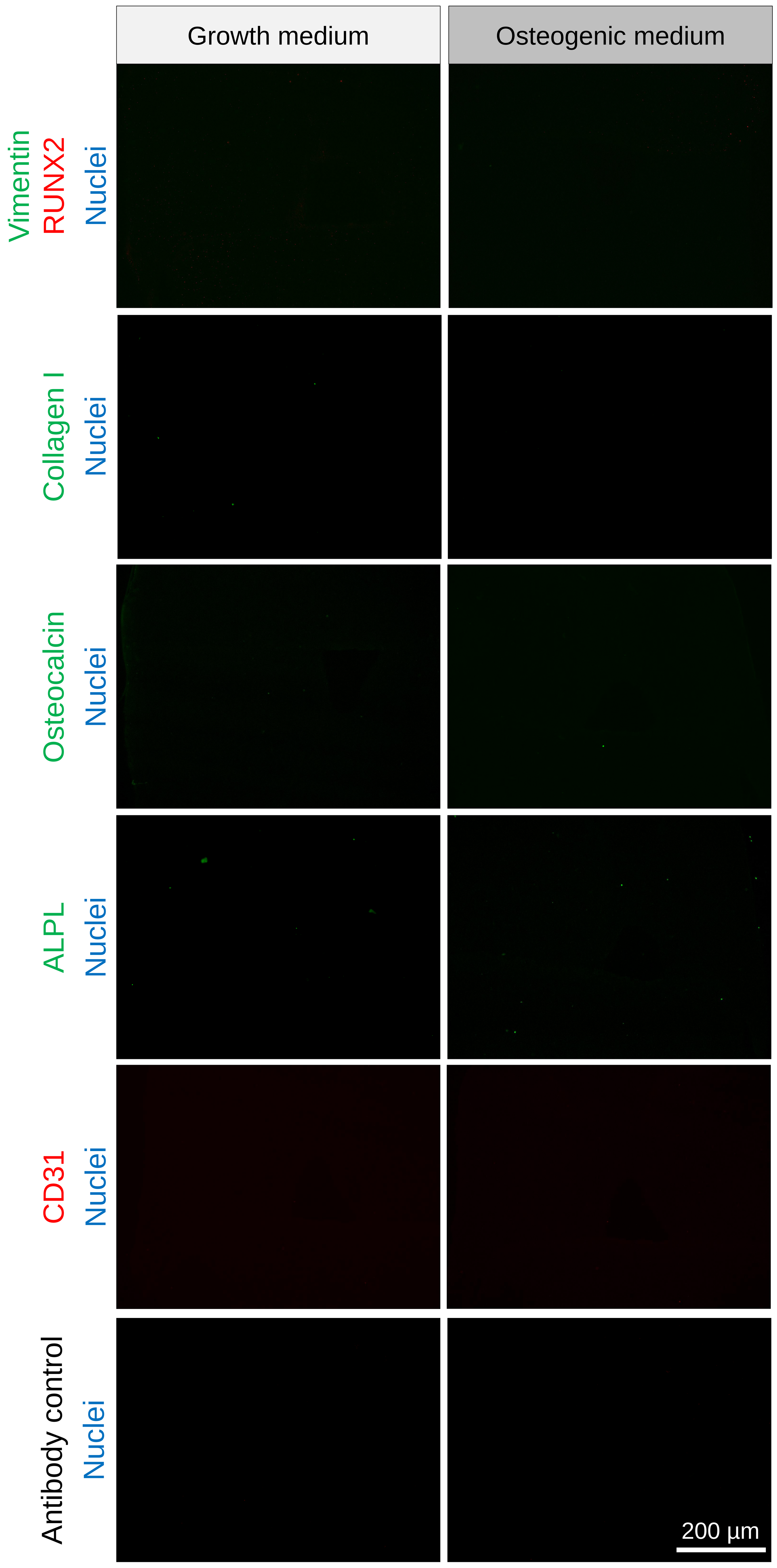

Supplementary Figure S2. Immunohistological staining for protein expression of osteoblast (RUNX2 (Runt-related transcription factor 2), collagen I, osteocalcin, ALPL (alkaline phosphatase)) and endothelial cell markers (CD31) of cell-free constructs after 28 days of cultivation in growth and osteogenic medium.

Supplementary Table 1. P values of the statistical analysis of real-time PCR results. O = JHOBs + empty channels. O+H = JHOBs + HUVECs. GM = growth medium. OM = osteogenic medium. Significant differences are marked in bold.

Two-way ANOVA with Tukey's multiple comparison test *ALPL*										
	Day 0: O	Day 0: O+H	Day 7: O GM	Day 7: O+H GM	Day 7: O OM	Day 7: O+H OM	Day 28: O GM	Day 28: O+H GM	Day 28: O OM	Day 28: O+H OM
Day 0: O	-	>0,9999	>0,9999	>0,9999	>0,9999	0,9996	**0,0359**	**<0,0001**	>0,9999	0,9639
Day 0: O+H		-	>0,9999	>0,9999	>0,9999	0,9997	**0,037**	**<0,0001**	>0,9999	0,9665
Day 7: O GM			-	>0,9999	>0,9999	0,9999	**0,0425**	**<0,0001**	>0,9999	0,9762
Day 7: O+H GM				-		>0,9999	**0,0474**	**<0,0001**	>0,9999	0,9822
Day 7: O OM					-	0,9996	**0,036**	**<0,0001**	>0,9999	0,9642
Day 7: O+H OM						-	0,1737	**0,0003**	>0,9999	>0,9999
Day 28: O GM							-	0,2651	0,0782	0,4332
Day 28: O+H GM								-	**0,0001**	**0,0013**
Day 28: O OM									-	0,9968
Day 28: O+H OM										-

One-way ANOVA with Tukey's multiple comparison test *DMP1*										
	Day 0: O	Day 0: O+H	Day 7: O GM	Day 7: O+H GM	Day 7: O OM	Day 7: O+H OM	Day 28: O GM	Day 28: O+H GM	Day 28: O OM	Day 28: O+H OM
Day 0: O	-	-	-	-	-	-	-	-	-	-
Day 0: O+H		-	-	-	-	-	-	-	-	-
Day 7: O GM			-	-	-	-	-	-	-	-
Day 7: O+H GM				-	-	-	-	-	-	-
Day 7: O OM					-	-	-	-	-	-
Day 7: O+H OM						-	-	-	-	-
Day 28: O GM							-	0,9629	>0.9999	0,9435
Day 28: O+H GM								-	0,9465	0,7014
Day 28: O OM									-	0,8421
Day 28: O+H OM										-

Two-way ANOVA with Tukey's multiple comparison test *COL1A1*										
	Day 0: O	Day 0: O+H	Day 7: O GM	Day 7: O+H GM	Day 7: O OM	Day 7: O+H OM	Day 28: O GM	Day 28: O+H GM	Day 28: O OM	Day 28: O+H OM
Day 0: O	-	>0,9999	0,952	0,9537	0,8167	0,9998	0,0673	**0,0009**	>0,9999	0,9983
Day 0: O+H		-	0,9721	0,9732	0,8666	>0,9999	0,0536	**0,0007**	>0,9999	0,9956
Day 7: O GM			-	>0,9999	>0,9999	0,9999	**0,0027**	**<0,0001**	0,9876	0,4952
Day 7: O+H GM				-	>0,9999	0,9999	**0,0028**	**<0,0001**	0,9882	0,4997
Day 7: O OM					-	0,9935	**0,0011**	**<0,0001**	0,9171	0,2919
Day 7: O+H OM						-	**0,0141**	**0,0002**	>0,9999	0,8871
Day 28: O GM							-	0,7697	**0,04**	0,3548
Day 28: O+H GM								-	**0,0005**	**0,0079**
Day 28: O OM									-	0,9881
Day 28: O+H OM										-

Two-way ANOVA with Tukey's multiple comparison test *RUNX2*										
	Day 0: O	Day 0: O+H	Day 7: O GM	Day 7: O+H GM	Day 7: O OM	Day 7: O+H OM	Day 28: O GM	Day 28: O+H GM	Day 28: O OM	Day 28: O+H OM
Day 0: O	-	>0,9999	0,9998	>0,9999	>0,9999	0,9817	0,6599	0,9825	0,9846	0,9601
Day 0: O+H		-	>0,9999	0,9889	>0,9999	>0,9999	0,9499	>0,9999	>0,9999	0,9998
Day 7: O GM			-	0,9815	0,9997	>0,9999	0,966	>0,9999	>0,9999	>0,9999
Day 7: O+H GM				-	>0,9999	0,8398	0,3562	0,8434	0,8524	0,7663
Day 7: O OM					-	0,9788	0,6446	0,9798	0,982	0,9551
Day 7: O+H OM						-	0,9993	>0,9999	>0,9999	>0,9999
Day 28: O GM							-	0,9992	0,999	0,9999
Day 28: O+H GM								-	>0,9999	>0,9999
Day 28: O OM									-	>0,9999
Day 28: O+H OM										-

One-way ANOVA with Tukey's multiple comparison test *SP7*										
	Day 0: O	Day 0: O+H	Day 7: O GM	Day 7: O+H GM	Day 7: O OM	Day 7: O+H OM	Day 28: O GM	Day 28: O+H GM	Day 28: O OM	Day 28: O+H OM
Day 0: O	-	-	-	-	-	-	-	-	-	-
Day 0: O+H		-	-	-	-	-	-	-	-	-
Day 7: O GM			-	-	-	-	-	-	-	-
Day 7: O+H GM				-	-	-	-	-	-	-
Day 7: O OM					-	-	-	-	-	-
Day 7: O+H OM						-	-	-	-	-
Day 28: O GM							-	0,8852	0,4173	0,4492
Day 28: O+H GM								-	0,8685	0,8558
Day 28: O OM									-	0,9995
Day 28: O+H OM										-

Two-way ANOVA with Tukey's multiple comparison test *SPARC*										
	Day 0: O	Day 0: O+H	Day 7: O GM	Day 7: O+H GM	Day 7: O OM	Day 7: O+H OM	Day 28: O GM	Day 28: O+H GM	Day 28: O OM	Day 28: O+H OM
Day 0: O	-	>0,9999	>0,9999	>0,9999	0,9981	>0,9999	0,0587	**0,0004**	>0,9999	0,9097
Day 0: O+H		-	0,9991	0,999	0,9818	>0,9999	0,1077	**0,0008**	>0,9999	0,9772
Day 7: O GM			-	>0,9999	>0,9999	>0,9999	**0,0171**	**0,0001**	0,9992	0,622
Day 7: O+H GM				-	>0,9999	>0,9999	**0,0168**	**0,0001**	0,9991	0,6178
Day 7: O OM					-	0,9995	**0,0072**	**<0,0001**	0,9833	0,3978
Day 7: O+H OM						-	**0,0449**	**0,0003**	>0,9999	0,8614
Day 28: O GM							-	0,6134	0,1046	0,7026
Day 28: O+H GM								-	**0,0008**	**0,0165**
Day 28: O OM									-	0,9753
Day 28: O+H OM										-

One-way ANOVA with Dunnett's multiple comparison test *ALPL*										
	Day 0: O	Day 0: O+H	Day 7: O GM	Day 7: O+H GM	Day 7: O OM	Day 7: O+H OM	Day 28: O GM	Day 28: O+H GM	Day 28: O OM	Day 28: O+H OM
Human jawbone	0,0567	0,0569	0,0573	0,0578	0,0567	0,0633	0,1002	0,1459	0,0622	0,0714

One-way ANOVA with Dunnett's multiple comparison test *COL1A1*										
	Day 0: O	Day 0: O+H	Day 7: O GM	Day 7: O+H GM	Day 7: O OM	Day 7: O+H OM	Day 28: O GM	Day 28: O+H GM	Day 28: O OM	Day 28: O+H OM
Human jawbone	0,7773	0,7312	0,1884	0,19	0,1214	0,4004	0,6279	0,1705	0,8314	0,9985

One-way ANOVA with Dunnett's multiple comparison test *DMP1*										
	Day 0: O	Day 0: O+H	Day 7: O GM	Day 7: O+H GM	Day 7: O OM	Day 7: O+H OM	Day 28: O GM	Day 28: O+H GM	Day 28: O OM	Day 28: O+H OM
Human jawbone	-	-	-	-	-	-	0,4363	0,436	0,2001	0,2005

One-way ANOVA with Dunnett's multiple comparison test *RUNX2*										
	Day 0: O	Day 0: O+H	Day 7: O GM	Day 7: O+H GM	Day 7: O OM	Day 7: O+H OM	Day 28: O GM	Day 28: O+H GM	Day 28: O OM	Day 28: O+H OM
Human jawbone	0,1073	0,1218	0,0579	**0,0395**	**0,0459**	0,0678	0,0941	0,073	0,0725	0,0764

One-way ANOVA with Dunnett's multiple comparison test *SP7*										
	Day 0: O	Day 0: O+H	Day 7: O GM	Day 7: O+H GM	Day 7: O OM	Day 7: O+H OM	Day 28: O GM	Day 28: O+H GM	Day 28: O OM	Day 28: O+H OM
Human jawbone	-	-	-	-	-	-	**0,0434**	0,065	**0,0363**	0,0592

One-way ANOVA with Dunnett's multiple comparison test *SPARC*										
	Day 0: O	Day 0: O+H	Day 7: O GM	Day 7: O+H GM	Day 7: O OM	Day 7: O+H OM	Day 28: O GM	Day 28: O+H GM	Day 28: O OM	Day 28: O+H OM
Human jawbone	0,0816	0,0838	**0,0273**	**0,0273**	**0,0259**	**0,0291**	0,0518	0,0706	**0,0323**	**0,0389**
